# Protein Disorder in Plant Stress Adaptation: From Late Embryogenesis Abundant to Other Intrinsically Disordered Proteins

**DOI:** 10.3390/ijms25021178

**Published:** 2024-01-18

**Authors:** An-Shan Hsiao

**Affiliations:** Department of Biochemistry and Metabolism, John Innes Centre, Norwich Research Park, Norwich NR4 7UH, UK; an-shan.hsiao@jic.ac.uk

**Keywords:** intrinsically disordered proteins (IDPs), stress response, disorder-to-order transition, biomolecular interaction, liquid–liquid phase separation (LLPS)

## Abstract

Global climate change has caused severe abiotic and biotic stresses, affecting plant growth and food security. The mechanical understanding of plant stress responses is critical for achieving sustainable agriculture. Intrinsically disordered proteins (IDPs) are a group of proteins without unique three-dimensional structures. The environmental sensitivity and structural flexibility of IDPs contribute to the growth and developmental plasticity for sessile plants to deal with environmental challenges. This article discusses the roles of various disordered proteins in plant stress tolerance and resistance, describes the current mechanistic insights into unstructured proteins such as the disorder-to-order transition for adopting secondary structures to interact with specific partners (i.e., cellular membranes, membrane proteins, metal ions, and DNA), and elucidates the roles of liquid–liquid phase separation driven by protein disorder in stress responses. By comparing IDP studies in animal systems, this article provides conceptual principles of plant protein disorder in stress adaptation, reveals the current research gaps, and advises on the future research direction. The highlighting of relevant unanswered questions in plant protein disorder research aims to encourage more studies on these emerging topics to understand the mechanisms of action behind their stress resistance phenotypes.

## 1. Introduction

Human activities have released large amounts of greenhouse gases into the atmosphere, causing climate change [1]. Global climate change is the main cause of abiotic and biotic stresses for plants that include flooding, drought, heat, cold, salinity, pests, and microbes [2]. These environmental stresses have greatly influenced plant physiological processes and have adverse effects on agriculture productivity [2,3]. To tackle the environmental stresses, sessile plants have evolved multiple processes including stress sensing, hormone regulation, signal transduction, gene expression, and regulatory pathways to adjust growth and development in a spatiotemporal manner [4,5]. Understanding the mechanism of plant stress adaptation to adverse environmental conditions will open new opportunities for agricultural applications and global food security.

Intrinsically disordered proteins (IDPs) are a group of proteins natively lacking defined three-dimensional structures. The peculiarities of their amino acid sequences are known to be depleted in order-promoting residues (tryptophan, cysteine, tyrosine, isoleucine, phenylalanine, valine, asparagine, and leucine) and enriched in disorder-promoting residues (arginine, proline, glutamine, glycine, glutamate, serine, alanine, and lysine) [6,7]. The amino acid biases contribute their exceptional spatiotemporal heterogeneity and low conformational stability, which drive their atypical response to changing environments [6,7]. IDPs can gain structures in the presence of various osmolytes, under the changes in temperature and pH, and perform a disorder-to-order transition when interacting with binding partners (i.e., cellular membranes, proteins, metal ions, and DNA) [6,7,8,9,10]. These features allow IDPs to sense environmental changes, mediate corresponding signalling pathways, and control and fine-tune plant metabolism in response to light, mechanical forces, pH, redox potential, and drought/salt concentration [11,12,13]. Thus, IDPs are thought to play critical roles in dynamic and plastic responses for the survival of sessile plants under the constantly changing environment [14,15,16].

The great importance of IDPs in stress adaptation is shown by extremophiles (i.e., anhydrobiotic tardigrades and resurrection plants) exhibiting extreme tolerance to various environmental stressors. Tardigrades survive in adverse environments via a set of highly disordered proteins that protect biomaterial through vitrification [17,18]. Two plants with relatively high protein disorder abundance, resurrection grass (*Oropetium thomaeum*) and switch grass (*Panicum virgatum*), are both stress-tolerant [19]. A proteomics analysis revealed that various IDPs were induced in the resurrection plant *Haberlea rhodopensis* during stress responses [20], whereas IDPs in wheat and barley are mainly involved in regulating cellular and biological processes in response to stress [21]. The function of IDPs in protecting biomolecules under stress conditions has been proposed [22], but the precise mechanisms of their action are still largely unknown.

Intrinsically disordered regions (IDRs) of transcription factors are highly adaptive and are proposed to provide functional versatility in molecular recognition via their binding plasticity [22,23,24]. IDRs can also serve as signalling hubs that regulate a diverse array of signal transduction pathways [9,25]. IDPs/IDRs are key triggers of liquid–liquid phase separation (LLPS) to form biomolecular condensates, which allow the spatiotemporal organization of biochemical reactions by concentrating macromolecules locally [26,27]. In animal cells, biomolecular condensates contribute to the biogenesis of macromolecular machineries essential for gene expression, the sequestration of specific factors to regulate cellular processes, and the interconnection of various diseases and innate immunity [28,29]. Recent studies revealed that plant biomolecular condensates driven by IDRs switch the defence programming through sequestration and regulate translation and protein quality control machineries in the plant immune response [30,31,32]. The study of LLPS is still an emerging area in plant research [33], and future research providing more mechanistic insights into how LLPS is involved in plant stress adaptation is expected.

This article summarizes the current knowledge of the physiological roles of plant IDPs in abiotic and biotic stress responses and discusses their potential molecular functions, highlighting the structure–function relationship of IDPs, interactions between IDPs and their partners, and the role of LLPS under various stress conditions. This article proposes the conceptual principles of how the structural flexibility of protein disorder leads to plant stress adaptation and pinpoints unanswered but relevant questions and the potential approaches to overcome the current limitation of plant IDP research.

## 2. Late Embryogenesis Abundant (LEA) Proteins Confer Abiotic Stress Tolerance

Plant seeds are desiccation-tolerant organs and physiologically similar to anhydrobiosis [34]. During the late stages of seed maturation, a group of IDPs known as LEA proteins are highly expressed in plant seeds before they enter the desiccation phase [35,36]. LEA proteins are thought to be involved in desiccation tolerance by modulating various clients [10,35,36,37]. LEA proteins are widely studied in model and crop plants such as rice, tomato, and Arabidopsis. There are 34, 60, and 51 *LEA* genes identified in rice [38], tomato [39], and Arabidopsis [40], respectively. *LEA* genes in plants are non-randomly distributed within the chromosomes, and segmental and tandem duplications drive *LEA* gene expansion in the genome during evolution [39,41,42].

Most *LEA* genes have elements responding to abscisic acid (ABA) and/or a low temperature in their promoter regions, such as ABA response elements (ABREs) and C-repeats (CRTs), which agrees well with *LEA* genes being induced by abiotic stresses such as drought, salinity, heat, and freezing in vegetative tissues [40]. Drought and salinity stresses trigger ABA signalling, whereas sucrose-nonfermenting-1-related protein kinases (SnRK2s) function upstream of the transcription factors ABA-INSENSITIVE 3 (ABI3), ABI5, and ABFs (ABA-responsive element-binding factors) to regulate the *LEA* gene expression via ABRE [43,44]. Cold stress activates calcium signalling and the mitogen-activated protein kinase cascade pathway, whereas CBFs (C-repeat binding factors) regulate the expression of the group 2 *LEA* dehydrins via CRT [44] (Table 1). Most LEA proteins are part of a more widespread group of proteins called “hydrophilins”, which are desiccation-related IDPs and have been discovered in archeal, eubacterial, and eukaryotic domains, including yeast, nematodes, tardigrades, and plants [34,45,46,47]. Hydrophilins are proposed to be stress effectors against desiccation in anhydrobiotes [48]; therefore, it is not surprising that LEA proteins are often involved in various stress responses such as desiccation resistance in plants [37,44,49].

There are several well-known examples of LEA proteins in stress responses. Arabidopsis dehydrins EARLY RESPONSE TO DEHYDRATION 10 (ERD10) and ERD14 function as chaperones to protect cells under a high salinity, drought, and low temperature [50]; LOW-TEMPERATURE-INDUCED 30 (Lti30) protects the membrane during cold and dehydration stress [51,52]; the group 3 LEA protein COLD-REGULATED 15A (COR15A) confers freezing tolerance by interacting with the membrane [53]; and legume physiologically mature (PM) proteins, which belong to various LEA groups, function in abiotic stress tolerance [54,55,56]. Their proposed mechanism is discussed in the following sections. Besides the reviews summarizing dehydrins in abiotic stress tolerance in various plant species [44,49], recent reports showed that heterologous expression of XsLEA1-8 from the monocot resurrection plant *Xerophyta schlechteri* [57], MsLEA-D34 from alfalfa (*Medicago sativa* L.) [58], and dehydrin CdDHN4 from bermudagrass (*Cynodon dactylon* × *Cynodon transvaalensis*) [59] increased osmotic and salt tolerance in Arabidopsis. These reports suggest that in general, stress-responsive LEA proteins confer stress tolerance among different plant species.

The analysis of LEA proteins in Arabidopsis, tomato, and orchid revealed their wide subcellular distribution [41,60,61]. Experimental data showed Arabidopsis LEA proteins localized to the cytosol, nucleus, mitochondria, plastid, ER, and pexophagosome [60]. Most of the tomato LEA proteins were predicted to target the nucleus or cytoplasm, with some in the mitochondria, chloroplasts, or extracellular matrix [41]. As well, subcellular localization prediction indicated that orchid LEA proteins are located in the nucleus, cytoplasm, chloroplasts, and mitochondria [61]. LEA proteins are proposed to protect the integrity of membranes and the activity of enzymes and biomolecules under stress conditions [22]. Therefore, their ubiquitous expression might provide protection to the corresponding membranes of various organelles as well as enzymes and sequestering targets localized in different cellular compartments under certain stress conditions [35,60]. Multiple cellular locations were also observed in another IDP, the Stress and Growth Interconnector (SGI), from the oil crop rapeseed (*Brassica napus*), which enhanced biomass and yield under drought conditions via multifaceted interactions with catalases and dehydrins [62]. A wheat IDP, the *Triticum aestivum Fusarium* Resistance Orphan Gene (TaFROG), showed spatial transition by changing its nucleus localization to cytosolic bodies when interacting with SnRK1 [63]. Thus, the spatial flexibility of IDPs implies their versatile functions in dual/multiple subcellular localizations.

Although more novel stress-responsive IDPs in plants are being investigated, LEA proteins are still the most-studied plant IDP family (Table 2). Despite overwhelming functional evidence suggesting that LEA proteins are widespread mediators of abiotic stress tolerance in plants, their mechanisms of protection are largely unknown. Speculation and preliminary studies suggested that LEA proteins could interact with vastly different target biomolecules during stress conditions, such as molecular shielding for enzyme protection, membrane stabilization via lipid binding, and interactions with various clients as described in the following sections.

**Table 1:** Hormone regulation of IDPs/IDRs mentioned in this article.

Hormone	Regulation of IDP/IDR Function in Stress Responses	Stressor Types	References
ABA	The expression of many *LEA* genes in plants is closely regulated by ABA and the corresponding signalling pathways. The promoter regions of *LEA* genes bear ABREs and CRTs, which can be recognized by various transcription factors such as ABI3, ABI5, ABFs, and CBPs during drought, salinity, and cold stress.	Abiotic stress	[43,44]
ABA	Rice RePRP is induced by water deficit and ABA in the root elongation zone and is sufficient and necessary for repression of root development by water deficit or ABA.	Abiotic stress	[64]
ABA, BR	Rice REM4.1 is transcriptionally upregulated by ABA and inhibits the formation and activation of a BR receptor kinase (BRI1-SERK1) complex, serving as a link between the ABA and BR signalling pathways.	Abiotic stress	[65]
SA	The major defence hormone SA triggers AtREM1.2/1.3-dependent membrane lipid nanodomain assembly, leading to plasmodesmata closure to impede virus spreading.	Biotic stress	[66]
SA	SA rapidly triggers the formation of nuclear GBPL3 condensates, which reprogram gene expression for disease resistance.	Biotic stress	[67]
SA	SA induces NPR1 condensates in cytoplasm to sequester and degrade stress proteins involved in cell death, promoting cell survival during the immune response.	Biotic stress	[30]
SA	SA induces massive formation of HEM1 condensates to restrict the availability of translation components and prevent immune gene translation during ETI.	Biotic stress	[32]

IDPs/IDRs, intrinsically disordered proteins/intrinsically disordered regions; ABA, abscisic acid; LEA, late embryogenesis abundant; ABREs, ABA response elements; CRTs, C-repeats; ABI3, ABA-INSENSITIVE 3; ABI5, ABA-INSENSITIVE 5; ABFs, ABA-responsive element-binding factors; CBPs, C-repeat binding factors; RePRP, repetitive pro-rich protein; BR, brassinosteroid; REM4.1, REMORIN 4.1; BRI1, brassinosteroid-insensitive 1; SERK1, somatic embryogenesis receptor-like kinase 1; SA, salicylic acid; GBPL3, guanylate-binding protein (GBP)-like GTPase 3; NPR1, NONEXPRESSOR-OF-PATHOGENESIS-RELATED GENE 1; HEM1, named for heme biosynthesis in the yeast homologue; ETI, effector-triggered immunity.

## 3. Molecular Shielding for Enzyme Protection

Many studies have shown that plant IDPs (mainly LEA proteins) are able to protect lactate dehydrogenase enzyme activity against damage caused by desiccation, heat stress, or freezing and thawing cycles [59,68,69,70,71,72,73,74,75,76]. The disorder and flexibility structure of LEA proteins may allow them to act as a kind of “molecular shield”, forming a physical barrier between neighbouring macromolecules and preventing their aggregation and inactivation under stress conditions [77]. LEA proteins can act sterically as noninteracting space fillers and/or interact with client proteins electrostatically via polar and charged amino acids. This molecular shield function is proposed to be distinct from classical molecular chaperones assisting protein folding via hydrophobic interactions and tight complex formation. However, both protect client targets against intramolecular damage [78]. Polyethylene glycols (PEGs) are similar to dehydrins, the group 2 LEA proteins, in terms of polar polymeric chains with no appreciable structure. Both dehydrins and PEGs showed size-dependent enzyme cryoprotection, which suggests that the disorder and polar nature along with the hydrodynamic radius of the dehydrin contribute to the molecular shield mechanism [79,80,81]. Group 5 LEA proteins are atypical because they contain a high content of hydrophobic residues and are proposed to be natively folded [45]. However, the group 5 LEA protein, *Medicago truncatula* PM25 (MtPM25), was proved to be intrinsically disordered and able to prevent aggregation of proteins during freezing, heating, and drying stress treatments [55]. Hydrophobic MtPM25 absorbs up to three-fold more water than MtEM6, a hydrophilic group 1 LEA protein. Although most LEA proteins showed hydrophilic features in their molecular shielding function, the case of MtPM25 showed that different LEA proteins could adopt various stress mechanisms [55]. Besides hydrodynamic volumes, LEA proteins also have an effect on three solvent properties of water (dipolarity/polarizability, hydrogen-bond donor acidity, and hydrogen-bond acceptor basicity), which are involved in one of the proposed in vitro protective mechanisms of these proteins [82]. Post-translational modification such as phosphorylation could regulate the function of LEA proteins by changing their conformations and ligand binding properties [83,84]. For example, phosphorylated salt-tolerance soybean PM18, a member of group 3 LEA proteins, confers better protection of lactate dehydrogenase in the freeze–thaw cycles than its un- or dephosphorylated form [56].

**Figure 1:**
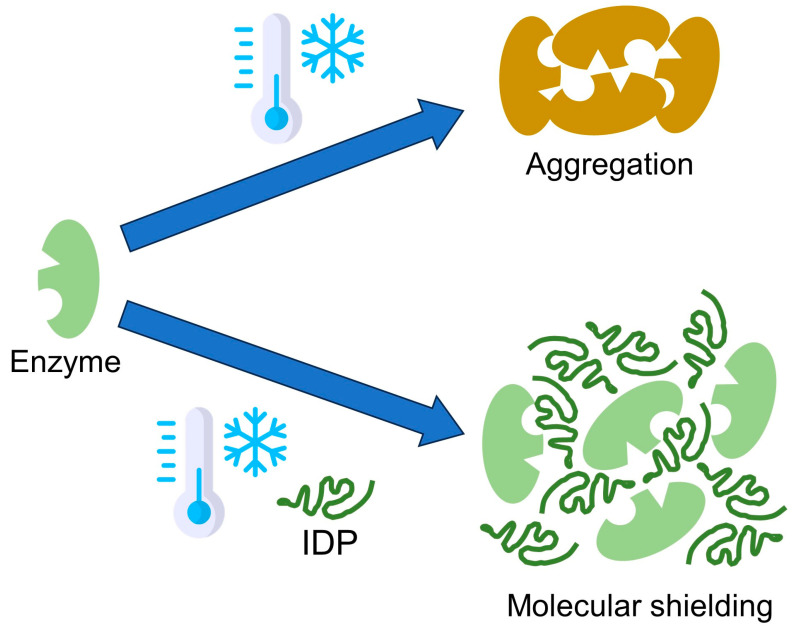
Putative molecular shielding mechanisms for cryoprotective activity of intrinsically disordered proteins (IDPs). During freezing stress, the enzyme (i.e., lactate dehydrogenase) increases protein–protein contacts to cause aggregation, which inactivates enzyme activity. In the presence of IDPs (i.e., LEA proteins) during freezing stress, the IDP acts as a shield, interfering with the interaction of the enzyme and keeping it in an active state.

Figure 1 illustrates plant IDPs protecting enzyme activity via a molecular shielding mechanism. The cryoprotection activity seems to not be correlated with specific amino acid sequences or the order of amino acid sequences but rather highly correlated with the biophysical properties of IDPs (i.e., hydrodynamic radius, the density of charged residues) [80,85]. Indeed, the scrambled grape dehydrin peptide showed cryoprotection of lactate dehydrogenase similar to that of the wild-type peptide [80]. Of note, randomly selected IDP fragments from the human genome, whose amino acid sequences are not related to plant dehydrins, also conferred substantial cryoprotection of lactate dehydrogenase [85]. This example emphasized the importance of the disorder nature in the cryoprotection effect. Hence, further examination of enzyme protection activity toward genome-wide plant IDPs will be critical in providing more evidence of their physicochemical properties related to their mechanisms of enzyme cryoprotection.

## 4. Disorder-to-Order Transition for Membrane Interaction

Membranes are often damaged during freezing and thawing or desiccation and rehydration. The rupture of the plasma membrane is one of the most commonly used indicators of cell death [86]. The group 2 LEA proteins, dehydrins, are often located near the membrane during abiotic stress [87,88,89,90]. They prevent the formation of oxidation-modified lipids and reduce the amount of electrolyte leakage generated by damaged membranes [91]. A recombinant citrus dehydrin prevented peroxidation of soybean liposomes in vitro, and overexpression of this citrus dehydrin enhanced cold tolerance in tobacco [92]. Dehydrins can alleviate oxidative damage in stressed plants by scavenging hydroxyl and peroxyl radicals or binding metals. The major residues in many dehydrins, glycine, histidine, and lysine, may be targets of these radicals [93,94,95]. Malondialdehyde is believed to be the final product of lipid peroxidation in the plant cell membrane. It is an important indicator of membrane system injury and disruption of cellular metabolism [96]. Malondialdehyde and electrolyte leakage in the freezing-tolerant loquat cultivar were found to be related to its higher accumulation of dehydrin proteins during freezing treatment [97]. Overexpression of LEA14 in foxtail millet reduced electrolyte leakage and enhanced salt and drought tolerance [98]. The inhibition of lipid oxidation and electrolyte leakage by LEA proteins and dehydrins help maintain membrane stability and fluidity; the mechanism involved may be found in the membrane binding properties in vitro.

**Figure 2:**
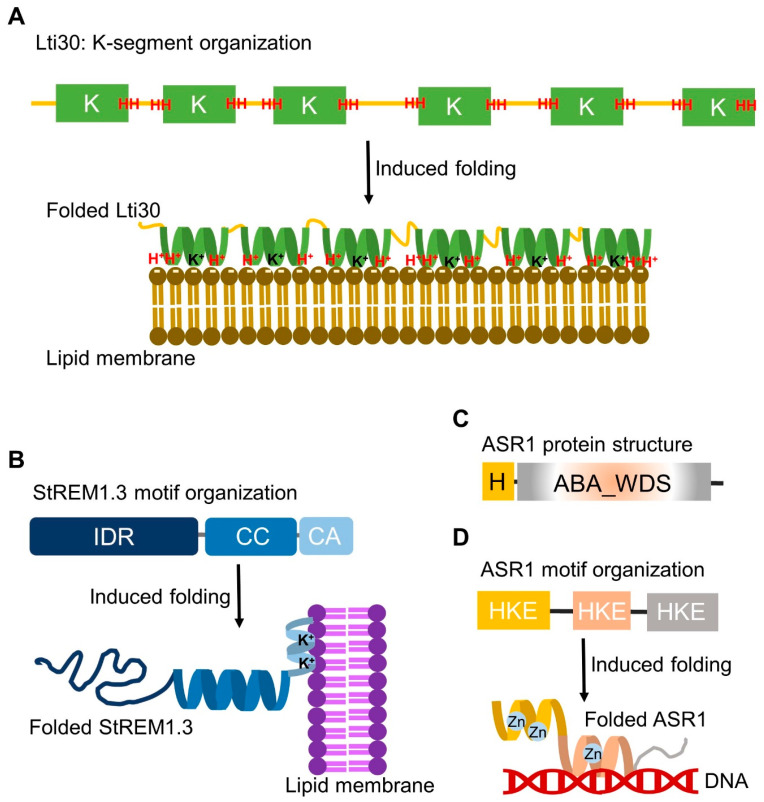
Motif organization and induced folding of three representative plant IDPs, Lti30, StREM1.3, and ASR1. (**A**) Arabidopsis Lti30 is a dehydrin that contains six K segments flanked by histidine (H)-rich sequences. K segments form helical conformations upon dehydration or when positively charged lysine (K^+^) and histidine (H^+^) interact with the negatively charged head groups of membrane phospholipids. (**B**) Potato remorin StREM1.3 contains the N-terminal IDR domain and a coiled-coil (CC) region and the C-terminal anchor (CA). The lipid-induced folding of CA region interacts with lipid polar heads via positively charged lysine (K^+^). (**C**) The protein structure of tomato ASR1 contains a short N-terminal consensus sequence comprising histidine (H) residues and a conserved abscisic-acid–water deficit stress (ABA_WDS) domain based on https://www.ebi.ac.uk/interpro/protein/UniProt/Q08655/ (accessed on 28 October 2023). (**D**) Disordered motif organization of tomato ASR1 drawn according to the sequences published on Goldgur et al. [106]. The three disordered motifs are rich in histidine, lysine, and glutamic acid (HKE). ASR1 can gain ordered structures upon the binding of zinc (Zn) at the N-terminal motif. Zn binding at the central motif contributes to the DNA-binding activity of ASR1. H^+^, positively charged histidine; K^+^, positively charged lysine; IDR, intrinsically disordered region.

The structural dynamics of dehydrins under stress conditions were summarized [99]. Studies regarding the in vitro interaction of dehydrins with model membranes revealed their disorder-to-order structural transition upon membrane binding [100,101]. This induced folding was shown by disordered K segments of dehydrins gaining ordered α-helix structures upon binding to anionic phospholipid vesicles [102] and micelles [100]. A good example is positively charged Arabidopsis dehydrin Lti30 binding to membranes by recognizing the negatively charged head groups of phospholipids [51] (Figure 2). This membrane interaction decreases the main lipid phase transition by 2.5 °C in vitro [51], corresponding to the decreased survival temperature of 3 °C observed in Arabidopsis overexpressing Lti30 [89]. A nuclear magnetic resonance (NMR) analysis suggested that disordered K segments of Lti30 locally fold into α-helical structures on the membrane surface [101] (Figure 2). Further studies showed that Lti30 may protect the cell by “cross-linking” the membrane lipids [52] and stabilizing the lamellar multilayer structure in response to changing water content [103]. Saltwater cress dehydrins showed induced folding [104], which induced temperature-dependent phase transitions and domain formation of lipid bilayers [105]. Thus, the IDP–membrane interaction may affect conformation changes as well as tertiary and quaternary associations.

IDPs can undergo a disorder-to-order transition and adopt ordered secondary structures such as α-helixes or β-sheets upon environmental changes (high temperature or extreme pH) and in the presence of their binding partners, artificial and natural membranes or osmolytes [6,107]. Besides dehydrins, various LEA proteins undergo the unfolding-to-folding transition to gain ordered conformations in the presence of artificial membranes [83]. Sodium dodecyl sulphate (SDS) is a widely used micelle-forming detergent for generating a membrane-like environment [108]. In the presence of SDS micelles, different groups of LEA proteins adopt α-helical structures; these proteins include AhDHN1 (group 2) from the halophytic species *Atriplex halimus* [109], pea LEAM (group 3) [110], GmPM1 and GmPM28 (group 4) from soybean [111], GmPM11 (group 1), GmPM6 (group 2), and GmPM30 (group 3) [112]. Other popularly used membrane mimetics are palmitoyl-oleoyl-phosphocholine (POPC) liposomes [113,114]. POPC liposomes induce secondary structure changes of the group 3 LEA protein Arabidopsis LEA7 [115] but not the group 4 LEA protein GmPM1 [54]. The group 3 LEA protein, Arabidopsis COR15A, folds into amphipathic α-helices upon dehydration or in the presence of high-concentration osmolytes [116,117]. COR15A interacts with POPC liposomes at the hydrophobic face of the amphipathic α-helix via hydrophobic interaction [118] and forms oligomeric assemblies that strongly depend on solution osmolarity [119]. The in vitro evidence leads to a hypothesis that COR15A suppresses the formation of non-lamellar lipids, the major cause of membrane damage upon dehydration, and stabilizes cellular membranes to confer freezing tolerance [53,117,118].

The above examples represent the flexible nature of IDPs, whose structural properties are regulated by the binding of a partner (i.e., membrane) as well as the environment (i.e., solution osmolarity). The disorder-to-order conformational change reflects an elegant mechanism of how IDPs might respond rapidly to changing environmental conditions such as stress treatments. Potential implications of the discussed IDP structural dynamics have been proposed as membrane stabilization to protect cells against water deprivation [83]. How conformational changes of IDPs when binding to membranes lead to retention of membrane fluidity and the suppression of membrane fusion events will be the next questions to be answered. Assigning in vivo meaning to in vitro evidence is still challenging.

## 5. Interactions around the Membrane

The preferred cell membrane localization of dehydrins supports their potential function in membrane protection, such as cold-acclimation-induced dehydrin localization near the cell membranes [88,89,90]. Cell membranes interact with IDPs and also provide an environment for IDPs to interact with their partners. A recent report from BiFC assays showed that pepper dehydrin DHN3 interacts with another dehydrin, HIRD11, most likely at the plasma membrane, but further evidence is required to address the role of this dehydrin–dehydrin interaction in ROS scavenging for drought and salt resistance [120]. The homodimeric and heterodimeric dehydrin–dehydrin interactions can be found in different subcellular distributions. The Arabidopsis dehydrins COR47 and ERD10 formed homodimers in the cytoplasm and RAB18 in the nucleus and cytoplasm [121]. For the moss dehydrin DHNA, the self-association dominates at cellular membranes and is enhanced by high-temperature stress [122]. Moreover, the Arabidopsis dehydrins COR47, ERD10, and RAB18 interact with the aquaporin PIP2B at the plasma membrane [123]. The detailed mechanism of dehydrin–dehydrin interactions and how these interactions affect the interaction of dehydrins with other partners (i.e., aquaporin) await discovery. Autophagy has been reported to regulate the heavy metal stress response [124] and salt [125] and desiccation tolerance via an anti-cell-death function and protein quality control system [20]. Of note, the dehydrin in *Medicago truncatula*, MtCAS31, interacts with the aquaporin MtPIP2;7 in the ER for autophagic degradation under drought stress [126]. The alfalfa dehydrin DHN1 interacts with two aquaporins, PIP2;1 and TIP1;1, at the plasma membrane [127], but the role of this interaction in controlling intracellular water content against dehydration stress and aluminium toxicity is unknown. Nevertheless, the interaction between dehydrins and aquaporins extends their function from membrane binding to the regulation of membrane proteins. Oligomerization of dehydrins in various cellular compartments may suggest that they have spatial regulation roles for interacting with corresponding membranes of various organelles as well as various partners. Indeed, LEA proteins in Arabidopsis, tomato, and orchid showed wide subcellular distribution [41,60,61].

Remorin is a plant-specific IDP family localized at the plasma membrane and thought to regulate membrane nanodomain assembly [128,129,130]. Potato remorin 1.3 (StREM1.3) is a case of disorder-to-order transition for membrane interaction [130]. The lipid-induced α-helical folding region of the unstructured StREM1.3 C-terminal anchor may interact with the lipid polar heads, whereas the hydrophobic region is embedded inside the lipid phase, forming a nanodomain organization [130]. In comparison to membrane-induced folding of Arabidopsis dehydrin Lti30, both Lti30 and StREM1.3 interact with lipid polar heads through electrostatic interactions via positively charged amino acids (lysine and histidine in Lti30 and lysine in StREM1.3; Figure 2). Although disordered K segments of Lti30 and the C-terminal anchor of StREM1.3 differ in primary amino acid sequences, both undergo lipid-induced folding into α-helical conformations for membrane interactions.

The study of *Medicago truncatula* remorin SYMREM1 suggested the scaffold role of remorins in regulating plasma membrane nanodomains during the symbiosis process: SYMREM1 stabilizes the interactions between FLOTILLIN 4 and LysM receptor-like kinase LYK3 at the plasma membrane during nodulation-promoting signalling [131]. The phosphorylation of remorins was modulated by biotic and abiotic stressors such as the bacterial “elicitor” peptide Flg22, oligogalacturonides mimicking wounding and herbivory, nitrogen deprivation, ABA, H_2_O_2_, cold, osmotic pressure, and salinity [132,133,134,135]. Perception of a virus leads to phosphorylation of group 1 remorins in *Solanaceae*, thus increasing plasma membrane mobility and facilitating the interaction with remorin-interacting proteins, causing an increase in callose deposition at the plasmodesmata and inhibiting virus cell-to-cell movement [130,136,137,138]. In Arabidopsis, AtREM1.2 and AtREM1.3 interact with general regulatory factor 10 (GRF10) to organize lipid raft nanodomains and participate in plasmodesmata closure to hamper virus spread [66]. The ABA-induced rice remorin OsREM4.1 was associated with OsSERK1 (somatic embryogenesis receptor-like kinase 1) at the plasma membrane, whereas the phosphorylation of OsREM4.1 dissociated the OsREM4.1–OsSERK1 complex; thus, OsSERK1 was able to interact with OsBRI1 (brassinosteroid-insensitive 1) to form the OsBRI1–OsSERK1 receptor kinase complex and activate brassinosteroid (BR) signalling: this process maintains a dynamic equilibrium between ABA and BR signalling [65]. There was a gain of function via the heterologous overexpression of mulberry MiREM1, foxtail millet SiREM6, and poplar PeREM6.5 in Arabidopsis-conferred resistance to salt stress [139,140,141], whereas PeREM6.5 was thought to enhance the plant’s ability to maintain ionic homeostasis under salinity by regulating H+-ATPase activity at the plasma membrane [141].

The disorder nature enables remorins to be an interaction hub scaffolding at the plasma membrane, interacting with various membrane proteins/receptors and thus playing a role in plant–microbe interactions and hormone signalling during stress responses [65,142]. Important topics to be investigated in the future are the functional versatility of remorin family members, consequences of the phosphorylation(s) of remorin-disordered regions in terms of conformational changes and protein–protein interactions, and the molecular mechanisms underlying remorin plasma-membrane–nanodomain organization under different stress conditions. These investigations will provide a deeper understanding of the remorin function in plant interactions with pathogens and symbionts, responses to abiotic stresses, and hormone signalling, which is useful for engineering and modifying remorin-mediated cellular processes for sustainable agriculture.

## 6. Metal Ion Binding Induced Folding of Stress-Tolerant ASR Proteins

Dehydrins and group 4 LEA proteins showed conformational changes and oligomerization when bound to metal ions, which may function in reducing oxidative damage induced by abiotic stress in plants [143,144,145]. Here, I discuss the group 7 LEA proteins, which correspond to the ASR (ABA, stress, and ripening) protein family that is conserved in many plant species but not found in Arabidopsis [45]. There are 5, 6, and 33 *ASR* genes in tomato, rice, and wheat, respectively [146,147,148]. Many *ASR* genes respond to ABA and abiotic stress such as drought, salt, and cold [146,149,150,151,152,153,154,155]. The ASR protein structure consists of a short N-terminal consensus sequence comprising histidine residues and a conserved ABA_WDS (water deficit stress) domain (pfam02496 and IPR003496) in the C-terminal region [156,157]. Heterogeneous expression of *ASR* genes often confers varying abiotic stress tolerance in different plant species. Overexpressed tomato and wheat ASR1 in tobacco plants increased salt and drought tolerance, respectively [153,158]; overexpression of lily ASR conferred drought and salt tolerance in Arabidopsis by increasing the expression of ABA/stress-regulated genes [159]; and overexpression of ASR from the extreme halophyte *Suaeda liaotungensis* conferred tolerance to salt, drought, and low temperatures in Arabidopsis [160]. Genetic studies revealed that *ASR1*-overexpressing tomato plants showed enhanced tolerance to water stress [146,158], and *OsASR1*-overexpressing rice showed enhanced salinity and drought tolerance [155], but the in vivo mechanism of how ASR proteins enhance stress tolerance is still largely unknown.

Similar to other LEA proteins, ASR proteins are intrinsically disordered. Biochemical and biophysical analyses of ASR proteins provided in vitro data hinting at the potential mechanism involved. Recombinant tomato ASR1 protein possesses zinc-dependent DNA-binding activity [161], which implies its transcription factor function as shown by tomato ASR1 targeting cell-wall-related and aquaporin genes [162]. In vitro studies of recombinant tomato ASR1 showed that ASR1 can adopt different conformations such as α-helix or polyproline type II in response to environmental changes, and the binding of zinc ions promotes α-helix folding and homodimerization of ASR1 [106,163,164]. Disordered segments of tomato ASR1 are enriched with histidine, lysine, and glutamate [106,163]. As compared with the induced folding of dehydrin K segments upon dehydration or membrane binding, the induced folding of tomato ASR1 is due to zinc binding via histidine residues [106,163] (Figure 2). Zinc-induced folding of ASR proteins was also observed in rice, barley, and wheat [165,166]. ASR proteins do not contain any of the known zinc-binding motifs described previously, such as the zinc finger, a common motif in DNA-binding proteins [167]. The abundance of histidine in ASR proteins potentially provides a hint of their possible ability to bind bivalent cations such as zinc [161,168], but direct binding evidence is still lacking. The structural plasticity of ASR proteins in gaining α-helix and β-strand conformations supports their versatile functions; however, the structure–function relationship in stress tolerance awaits investigation. Current data revealed that metal ion binding to ASR proteins induces their folding, but how this conformational change relates to their transactivation capabilities remains unclear. We do not know whether ASR proteins play a role as a chromatin protective protein during stress or as a transcription activator in the stress signal pathway. Future studies using chromatin immunoprecipitation assays will help answer this question.

## 7. Ectopic IDP Expression Confers Stress Tolerance in Yeast and Bacteria

Stress tolerance phenotypes have been observed in ectopic expression of some plant IDPs in unicellular organisms such as *Escherichia coli* (*E. coli*) and yeast. The heterologous hydrophilic domain of LEA expression conferred enhanced desiccation and oxidation tolerance to *E. coli* [169]. Arabidopsis ERD14 could protect *E. coli* cells under heat stress [170,171]. Overexpression of cucumber (*Cucumis sativus*) dehydrin CsLEA11 in *E. coli* enhanced cell viability and conferred tolerance to heat and cold stress [42]. The expression of eight Arabidopsis LEA genes enhanced desiccation tolerance in yeast [172]. In vivo expression of XsLEAs from the monocot resurrection species *Xerophyta schlechteri* enhanced *E. coli* viability under salt, osmotic, and heat stress [57]. Recombinant AcLEA function was evaluated using *E. coli* as an in vivo model, showing an important protection role against desiccation, oxidant conditions, and osmotic stress [173]. The moss dehydrin DHNA provides stress tolerance to *E. coli* cells via proteome protection mediated by D-segments [122]. Heterologous expression of ABA_WDS domains from barley and wheat ASR proteins in the yeast *Saccharomyces cerevisiae* improved its tolerance to salt, heat, and cold stresses [168].

Ectopic expression of plant LEA proteins in unicellular organisms such as *E. coli* and yeast conferring stress tolerance is probably due to the widely distributed properties of hydrophilins across kingdoms. However, plant IDPs other than hydrophilins also confer stress tolerance in unicellular organisms. For example, a plant-specific DNA binding with one finger (DOF) transcription factor, rice DOF27, is intrinsically disordered and is implicated in the thermotolerance response in yeast [174]. A member from the *Poaceae*-specific RICE SALT SENSITIVE 1 (RSS1) family, unstructured wheat RSS1-Like 1, enhanced stress tolerance of Arabidopsis seedlings and yeast cells [175,176]. Therefore, ectopic expression of plant IDPs in protecting cellular matter across multiple kingdoms of life may not simply be attributed to the effect of the homology. The principle of physiochemical properties governing plant IDPs underpinning their quick sensitivity and responsiveness to changing environments and dynamic conformations to interact with various partners for stress tolerance needs to be investigated. Given that tardigrade disordered proteins are proposed to undergo LLPS and interact with various partners for transitioning into an anhydrobiotic status under extreme conditions [177], LLPS is potentially a way for plant IDPs to change the physiological status in different organisms to achieve an anhydrobiosis-like status for stress resistance.

## 8. Roles of LLPS in Plant Stress Responses

Disordered proteins have phase transition properties for undergoing LLPS and forming membrane-less biomolecular condensates. These properties enable a rapid assembly, disassembly, and concentration of cellular components and facilitate the dynamic formation of local reaction centres with spatiotemporal specificity [27,178]. Notably, LLPS is also involved in plant developmental phase transition such as flowering and seed germination [11]. The FLOWERING CONTROL LOCUS A (FCA) nuclear body driven by highly disordered FLX-LIKE 2 (FLL2) plays a role in transcriptional regulation to control flowering [179]. Stress granules are among the well-studied membrane-less condensates transiently assembled via LLPS in response to stress [180,181], whereas LLPS is induced when plants encounter various abiotic stresses such as a high temperature (35 °C and 37 °C) [182,183], a low temperature (4 °C) [184], salinity (0.4 M NaCl treatment) [185], and ROS (1 mM H_2_O_2_) [186]. LLPS driven by the IDR of phytochrome B (phyB) plays a major role in temperature sensing and thermomorphogenesis, suggesting an emerging mechanism for plants to directly respond to thermal changes [187]. A plant-specific prion-like protein was named FLOE1, the definition of floe being “a sheet of floating ice”, which is a phase-separated body of water [188]. Upon hydration, LLPS of intrinsically disordered FLOE1 allowed the embryo to sense water stress and promoted seed germination [188]. The importance of LLPS in the osmotic stress response was shown by the Arabidopsis transcriptional regulator SEUSS (SEU). The IDR of SEU is responsible for forming liquid-like nuclear condensates, which is indispensable for osmotic stress tolerance [189]. Unlike the well-established mechanism of FCA nuclear condensates in transcriptional control of flowering, we do not know whether the components of SEU nuclear condensates contain multiple active transcription regulators for promoting the transcription of osmotic stress-responsive genes.

Besides the aforementioned abiotic stress responses, LLPS is also involved in the biotic stress response as an interacting hub in plant immunity. LLPS is induced by bacterial phytopathogens *Pseudomonas syringae* pv. *Maculicola* ES4326 and *P. syringae* pv. tomato DC3000 as shown by the case of guanylate-binding protein (GBP)-like GTPases (GBPLs) [67]. The IDR of GBPLs is required for the assembly of LLPS-driven condensates within the nucleus and they are called GBPL defence-activated condensates, which coordinate defence-gene transcription for disease resistance [67]. The IDR of the salicylic acid receptor NONEXPRESSOR-OF-PATHOGENESIS-RELATED GENE 1 (NPR1) forms cytoplasmic condensates and serves as an interacting hub to sequester and degrade proteins involved in programmed cell death in infection-adjacent cells with a low pathogen load [30]. LLPS of NPR1 plays a role in switching cellular programming from effector-triggered immunity (ETI) in infected cells to systemic acquired resistance in adjacent cells [30]. A recent report highlighted the importance of LLPS in regulating translational reprogramming in plant immunity [32]. Arabidopsis HEM1 (named for heme biosynthesis in the yeast homologue) contains a plant-specific IDR called the low-complexity domain, which undergoes LLPS to form condensates to interact with translation factors, thus suppressing translation efficiency of the pro-death immune genes during ETI [32]. Although the interaction of HEM1 with NPR1 in the cytoplasmic condensates has not been detected [32], both cases suggest a common role of LLPS in balancing tissue health and disease resistance during the plant immune response.

Protein disorder has a versatile contribution to both the plant immune system and pathogen virulence [190,191]. The N-terminal IDR of prosystemin, the 200-aa precursor of the peptidic hormone systemin responding to wounding and herbivore attack, was able to induce defence-related genes and protect tomato plants against *Botrytis cinerea* and *Spodoptera littoralis* larvae [192]. In the plant defence response, RPM1-INTERACTING PROTEIN 4 (RIN4) is a negative regulator and targeted by multiple bacteria effectors [193,194,195]. The IDR of RIN4 was known to fold various conformations when interacting with different partners such as bacterial effectors and be under regulation of post-translational modification [196,197,198,199]. The latest report suggested that the intrinsically disordered nature of RIN4 provides a flexible platform to broaden pathogen recognition specificity by establishing compatibility with otherwise incompatible leucine-rich repeat immune receptor proteins [200,201]. Besides its involvement in plant immunity responses, LLPS is involved in pathogen virulence. The IDR of FgRpd3 (reduced potassium dependency 3) from wheat head blight fungus *Fusarium graminearumis* undergoes LLPS, which assists its interaction with inhibitor of growth (ING) proteins for regulating histone deacetylation and gene expression, thus affecting fungal development and pathogenicity [202].

Achieving the necessary spatiotemporal resolution to deduce the parameters that govern the assembly and behaviour of LLPS is challenging [203], especially when it involves the dynamics and multiple subcellular locations of IDPs (i.e., LEA proteins). Therefore, as compared with extensive studies of plant LEA proteins in stress tolerance, much less research has investigated plant LEA proteins promoting LLPS [204]. Biomolecular condensates formed by LEA proteins from the anhydrobiotic animal brine shrimp were considered to act as protective compartments for desiccation-sensitive proteins to promote dehydration tolerance during anhydrobiosis [205,206]. Whether LLPS has a similar role in plant LEA protein functioning in stress tolerance still awaits investigation. Despite recent discoveries of novel plant IDPs involved in LLPS to generate biomolecular condensates during stress sensing and signalling, several questions remain. What are the determining factors of LLPS for forming biomolecular condensates (i.e., specific amino acid sequences of IDPs, specific IDP conformation, or biophysical properties)? And what are the components of biomolecular condensates in various stress situations? Future investigation dissecting the spatial and temporal rules guiding biomolecular condensate formation under various stress conditions is important to understand the roles of plant IDP-driven LLPS in stress responses.

## 9. IDPs Interacting with the Cytoskeleton for Stress Adaptation

Changes at the cytoskeleton protein level and dynamics were observed upon cold [207], drought [208], and heavy metal [209] stress [210]. Depolymerization of actin filaments may prevent expansion-induced cell lysis during freeze−thaw cycles [211], and the cytoskeleton was thought to take centre stage in abiotic stress responses and resilience [212]. The rapid remodelling of the cytoskeleton can coordinate the movement of intracellular organelles, formation of immune microdomain complexes, transportation of defence compounds and turnover of recognizing receptors, cell-wall-based defence upon pathogen attack, as well as hormone-mediated cell expansion and division under abiotic stress [213,214,215,216,217]. Many cytoskeleton-associated proteins are IDPs or possess extensive IDRs [218,219]. The disordered nature of these cytoskeleton-associated proteins enables them to regulate the dynamics and organization of highly ordered cytoskeleton proteins for quick responsiveness to the changing environment. Dehydrins are thought to be able to stabilize the cytoskeleton under stress conditions, as shown by dehydrins from *Thellungiella salsuginea* facilitating the polymerization of actin filaments [211]. Recent findings highlighted the importance of IDPs in interacting with the cytoskeleton for stress adaptation in plants. Rice intrinsically disordered repetitive pro-rich protein (RePRP) interacts with both the actin and tubulin cytoskeleton to generate short-but-heavy roots under a water deficit [64]. Arabidopsis COMPANION OF CELLULOSE SYNTHASE1 (CC1) controls microtubule bundling and dynamics to sustain plant growth under salt stress via its intrinsically disordered N-terminus, which can diffuse bidirectionally along the microtubule lattice [220], similar to the mammalian tubulin-associated unit Tau [221]. As compared with the well-established mechanism of disordered Tau-microtubule interaction causing neurodegeneration, how protein disorders regulate cytoskeletal dynamics in plant stress responses is unclear. We still lack studies investigating why protein disorder interacting with the cytoskeleton results in neuron degenerative disease in mammals but stress adaptation in plants. Treatment with the actin depolymerizing drug latrunculin B can destabilize the association of SYMREM1 to the plasma membrane nanodomains [131], so the cytoskeleton may be involved in remorin scaffolding. In fact, both rice and Arabidopsis remorins interact with the actin cytoskeleton in plants [222,223,224]. The IDR of Arabidopsis remorin1.2 orchestrates high-order self-assembly and formin clustering to mediate a nanocluster of membrane nanodomains for actin remodelling during innate immune responses [224]. Such molecular condensation-regulated cytoskeleton remodelling has been identified in the mammalian system [225]. The similarity and differences of protein disorder mediating cytoskeleton dynamics between plant and animal systems need to be further investigated. Future research investigating the mechanism of molecular condensation regulating the cell wall–plasma membrane–cytoskeleton continuum during plant immune responses will answer the question of how protein disorder affects stress sensing and signalling. Because of the continuing race of disorder between plant hosts and pathogens, not surprisingly, a bacteria effector hijacks the host actin cytoskeleton, as shown by the *Xanthomonas* effector XopR affecting actin nucleation, crosslinking actin filaments and actin depolymerization in host cells via IDR-mediated LLPS [226]. Thus, IDP/IDR-mediated cytoskeleton dynamics are a double-edged sword in the biotic stress response in that they can affect membrane nanodomain distribution for plant defence (e.g., the case of remorins) and can be a hijacked target of pathogen infection.

## 10. Conclusions and Perspectives

**Figure 3:**
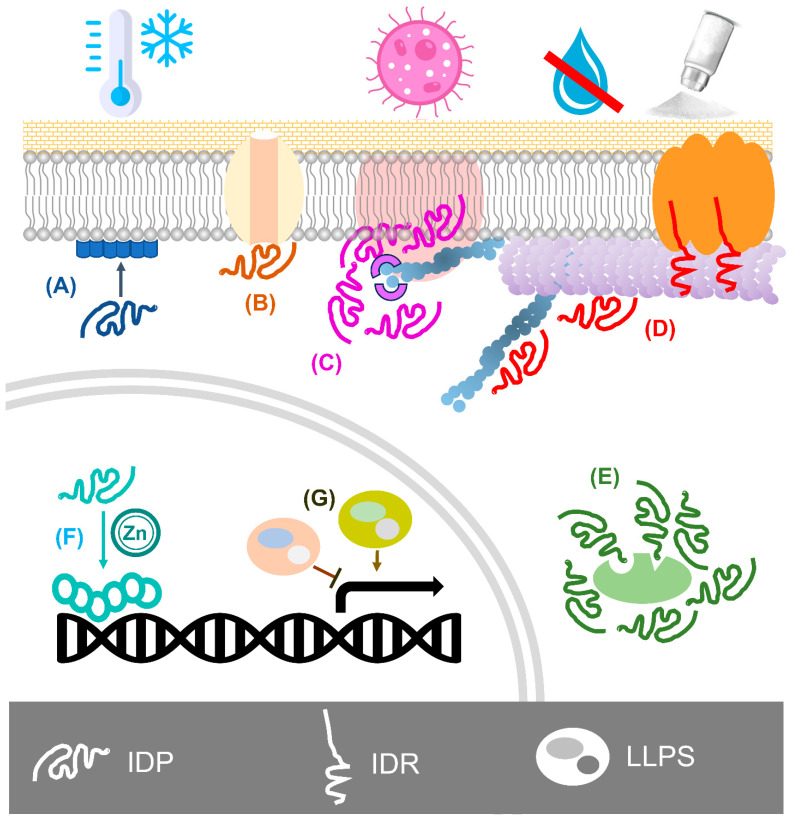
Protein disorder is important in abiotic and biotic stress resistance in plants. (**A**) Intrinsically disordered proteins (IDPs) can undergo disorder-to-order transition and form an α-helix to bind cellular membranes, maintaining membrane stability under freezing stress. (**B**) IDPs can interact with membrane proteins such as transporters to mediate their functions under stress conditions. (**C**) During plant–pathogen interactions, IDPs mediate the molecular condensation of the cell wall–plasma membrane–cytoskeleton continuum for immunity signalling transduction. (**D**) IDPs and intrinsically disordered regions (IDRs) interact with the cytoskeletal actin filaments and microtubules for adaptive growth under water deficit and salinity stress. (**E**) IDPs can form a molecular shield to protect enzymes under stress conditions. (**F**) Metal ion binding of IDPs induces their conformational changes and facilitates their interaction with DNA. (**G**) Liquid–liquid phase separation (LLPS) of IDPs may regulate gene expression during plant immunity responses.

The plasticity of IDPs is an easy and fast way for sessile plants to introduce versatility into protein interaction networks and quickly and efficiently adapt to environmental changes [227]. The ability of IDPs to interact with various partners gives them versatile functions in different cellular processes at various subcellular compartments during stress (Figure 3). With their multiple roles explored, the next step is to apply modern technologies in cell biology and biophysics for understanding the underlying mechanism [228,229,230,231,232]. Bioimaging tools are useful for studying plant physiological processes in vivo [233]. A recent report suggested that fixation causes artifacts of LLPS in the animal system [234], so live-cell imaging could be a preferable approach to dissect the phenomenon of LLPS. Single-particle tracking (SPT) is a valuable method for directly measuring and calculating the diffusion and dynamics within biomolecular condensates [203]. A recent study using SPT in yeast cells revealed the mechanism of mobility of RAD52 (named radiation-sensitive) within repair foci at DNA double-stranded break events and suggested that LLPS was required for DNA repair [235]. In plants, an SPT method has been developed for studying the distribution and dynamics of the plant membrane protein aquaporin [236]. Although not reported so far, using SPT with super-resolution microscopy is expected to dissect the movement of plant IDPs and the spatial and temporal regulation of LLPS (i.e., the formation of biomolecular condensates) during stress responses by directly “observing” how they diffuse in living cells in front of our eyes.

Of note, the fluorescence tags used in live-cell technology may alter LLPS and IDP properties, potentially causing artifacts [237,238]. Therefore, biophysics approaches can be complementary to bioimaging tools for overcoming these obstacles. Biophysics characterization such as NMR spectroscopy can describe the binding mechanisms and map the conformational dynamics of IDPs [228,239], and in-cell NMR is a promising solution to provide their structural information in living cells [240]. A preliminary study using in-cell NMR in bacteria showed the disordered nature of Arabidopsis dehydrin ERD14 in the cytoplasm of *E. coli* [170]. Future research to investigate the conformational changes in ERD14 upon stress treatments via in-cell NMR will help answer the question of how ectopic expression of plant IDPs confers stress tolerance in bacteria. In plants, the current bottleneck of in-cell NMR is the delivery of isotope-labelled proteins into living plant cells, but exogenous agents (bacteria and viruses) and cell-penetrating peptides could be used for eliminating the bottleneck [241]. Taken together, future research should use multidisciplinary approaches, especially combined with cell biology and biophysics tools, to catch the dynamic motions of plant IDPs both in vitro and in vivo.

Plant stress-responsive IDPs can have broad application beyond plant research, based on basic research of IDPs in plant stress responses as mentioned previously. A recent innovation showed that a FRET biosensor can monitor rapid intracellular changes under osmotic stress by utilizing the environmental sensitivity and structural plasticity of Arabidopsis LEA4-5 protein [242]. A better understanding of the biochemical properties and conformational information of plant IDPs under various stress conditions will provide the knowledge needed for programming, such as the amino acid sequence and responsiveness profiling. We should be able to produce synthetic peptides based on plant IDPs for different applications. For example, LEA proteins have promising cryoprotection properties, preserve desiccated biomaterials, and can act as anti-osmotic agents, which leads to novel opportunities in biomedical and pharmacological development [243].

With an increasing human population, food security is certainly one of the most serious global challenges today. Because IDPs have multiple functions in protecting plants under various abiotic and biotic stress conditions, plant stress-responsive IDPs have potential application as genetic engineering targets for generating stress-resistant crop plants. A potential future research direction is specifically targeting stress-responsive LLPS processes in crop plants to achieve temporary stress resistance during environmentally harsh periods. Understanding the mechanisms used by these IDPs that allow them to mediate abiotic and biotic tolerance in plants will not only close the knowledge gap but also reveal insights that may revolutionize crop resilience. Using multidisciplinary technologies to unravel the intricate workings of IDPs in plant stress adaptation will pave the way for the development of more robust and sustainable crops to solve food security problems.

## Figures and Tables

**Table 2:** Studies of late embryogenesis abundant (LEA) proteins mentioned in Section 2.

Plant Species	Finding	Topics	References
Rice (*Oryza sativa*)	Identification of 34 rice *LEA* genes and their transcript analysis under untreated, abscisic acid, osmotic, and salinity stress conditions.	Genomics	[38]
Tomato (*Solanum lycopersicum*)	Identification of 60 tomato *LEA* genes and their transcription and expression patterns in various tissues and under abiotic stress and phytohormone treatments. Duplication event in evolution of the *LEA* family.	Genomics	[39,41]
	Characteristics of the LEA proteins including disorder tendency and localization.	Proteomics	[41]
Arabidopsis (*Arabidopsis thaliana*)	Identification of 51 Arabidopsis *LEA* genes and their transcription patterns at different developmental stages, in different plant organs, and under different stress and hormone treatments. Respective promoter elements induced by abiotic stress.	Genomics	[40]
	Classification of Arabidopsis LEA proteins and their subcellular localization.	Cell biology	[60]
60 plant species ranging from green algae to angiosperms	Comprehensive synteny and phylogenetic analyses of *LEA* genes across 60 complete plant genomes showed their evolution and diversification.	Genomics	[42]
Orchid (*Dendrobium officinale*)	Identification of *Dendrobium officinale LEA* genes and their expression patterns under abiotic stress treatments. Classification of DfLEA proteins and their predicted subcellular localization.	Genomics	[61]
*Xerophyta schlechteri*	Enzyme protection function of XsLEA proteins under stressful conditions. Expression of XsLEAs increased abiotic stress tolerance in *E. coli* and Arabidopsis.	Stress physiology	[57]
Alfalfa (*Medicago sativa*)	Expression of *Medicago sativa* MsLEA-D34 increased plant tolerance to osmotic and salt stresses and caused Arabidopsis early flowering under drought and well-watered conditions.	Stress physiology	[58]
Bermudagrass (*Cynodon dactylon*)	Expression of *Cynodon dactylon* CdDHN4-L and CdDHN4-S increased salt tolerance in Arabidopsis, enzyme protection function, and disordered character.	Stress physiology	[59]
Common wheat (*Triticum aestivum*), durum wheat (*T. durum*), barley (*Hordeum vulgare*)	A comparison of dehydrins in common wheat, durum wheat and barley in the context of expression patterns at transcript and protein levels and their possible functions when exposed to various abiotic stress factors.	Stress physiology	[15]
Various	A summary of structural characterization of plant LEA proteins and their binding modes.	Structural biology	[22]
Various	A summary of plant LEA proteins from characterization to their functions in stress responses.	Stress physiology	[35]
Various	A summary of LEA functions during seed maturation and seed desiccation tolerance.	Stress physiology	[36]
Arabidopsis (*Arabidopsis thaliana*)	A discussion of LEA functions in stabilizing membranes or sensitive enzymes during dehydration.	Stress physiology	[37]
